# Effect of host-cell interactions on clonogenic carcinoma cells in human malignant effusions.

**DOI:** 10.1038/bjc.1980.131

**Published:** 1980-05

**Authors:** R. N. Buick, S. E. Fry, S. E. Salmon

## Abstract

We have studied the clonogenic capacity of tumour cells in agar from 38 malignant effusions from 31 patients with epithelial tumours. Colony formation of unfractionated cells, varies considerably from patient to patient, and is positively correlated with the percentage of tumour cells in the sample. Clonogenicity was shown to be reduced in 8/9 cases by removal of plastic-adherent and iron-phagocytic cells. In the ninth case, increased clonogenicity occurred after this procedure. When the autologous adherent cells were removed from the effusion and used in reconstitution experiments as an underlayer in a two-layer agar system, they were able to reverse the effect of the initial fractionation in a dose-dependent fashion. This indicates cellular communication based on release of a diffusible product of plastic-adherent cells. Morphological, phagocytic and prostaglandin-synthetic analysis of the fractions involved in the reconstitution experiments implicate the macrophage as the operative cell in this interaction. However, an accessory role for lymphoid cells or tumour cells themselves cannot be excluded.


*Br. J. Cancer* (1980) **41**, 695

### EFFECT OF HOST-CELL INTERACTIONS ON CLONOGENIC CARCINOMA CELLS IN HUMAN MALIGNANT EFFUSIONS

R. N. BUICK, S. E. FRY AND S. E. SALMON

*From the Section of Hematology and Oncology Department of Internal Medicine and The Cancer Center, University of Arizona College of Medicine, Tucson, Arizona*

Received 5 November 1979 Accepted 16 January 1980

**Summary.—We have studied the clonogenic capacity of tumour cells in agar from 38 malignant effusions from 31 patients with epithelial tumours. Colony formation of unfractionated cells varies considerably from patient to patient, and is positively correlated with the percentage of tumour cells in the sample. Clonogenicity was shown to be reduced in 8/9 cases by removal of plastic-adherent and iron-phagocytic cells. In the ninth case, increased clonogenicity occurred after this procedure. When the autologous adherent cells were removed from the effusion and used in reconstitution experiments as an underlayer in a two-layer agar system, they were able to reverse the effect of the initial fractionation in a dose-dependent fashion. This indicates cellular communication based on release of a diffusible product of plastic-adherent cells. Morphological, phagocytic and prostaglandin-synthetic analysis of the fractions involved in the reconstitution experiments implicate the macrophage as the operative cell in this interaction. However, an accessory role for lymphoid cells or tumour cells themselves cannot be excluded.**

HUMAN MALIGNANT EFFUSIONS contain a complicated mixture of cell types, including tumour cells, mesothelial cells, lymphocytes, macrophages and granulocytes (Light *et al.*, 1973). Such effusions have long provided biologists with convenient access to a cell suspension of human tumour tissue, and a number of epithelial tumour-cell lines have been derived from this source. The biological significance of the cell interactions occurring in malignant effusions are, however, relatively obscure. Tumour–lymphoreticular interaction has been studied extensively from the point of view of description and of specific cytotoxicity, both in animal and human tumours (Holden *et al.*, 1976; Wood *et al.*, 1975; Underwood, 1974; Evans, 1976). In addition, a number of reports have pointed to the fact that under certain circumstances there may be lymphoreticular stimulation of tumour growth (Norbury, 1977; Mantovani *et al.*, 1979; Fidler *et al.*, 1974). Indeed, Prehn (1977) has proposed a "lymphodependent phase of tumor growth" and Salmon & Hamburger (1978) have hypothesized that tumour growth was macrophage-dependent, partly on the basis of studies of depletion of phagocytic cells from ovarian carcinoma effusions (Hamburger *et al.*, 1978). In the present study, we have examined the stoichiometry of the cellular interactions with the perspective that tumours are examples of cell-renewal systems in which the majority of the cell population is maintained by the function of a few proliferative renewing cells (stem cells) (Steel, 1977) and may respond to growth stimulants or inhibitors from other cell populations. Tumour stem cells are considered to be critical in terms of

Reprint requests to: Ronald N. Buick, Ph.D., Ontario Cancer Institute, 500 Sherbourne Street, Toronto, M4X 1K9, Canada.

determination of response to treatment (Steel, 1977) and as such are the important cells to study from the point of view of putative host-cell–tumour interaction. One approach to assessment of tumour stem cells is the recent application of two-layer agar techniques to the assessment of clonogenic tumour cells (Hamburger *et al.*, 1978; Hamburger & Salmon, 1977; Buick *et al.*, 1979*a*). This paper describes the application of fractionation and reconstitution experiments to the study of controlling influences on tumour clonogenicity from cells derived from malignant effusions, and provides evidence of major interaction between reactive host cells and the clonogenic human tumour cells.

FIG. 1.—Relationship between number of cells adherent and number of cells plated. Effusion cells were plated at the concentrations shown as described in the Methods section. Results are expressed as mean ± s.e. of triplicate plates. (Pt 12a).

### METHODS

*Patients.*—Pleural or ascitic fluid (200–8000 ml) was obtained by paracentesis into heparinized (10 u/ml) vacuum bottles from 31 patients with histologically proven epithelial cancers (17 ovarian, 8 breast, 3 colon, 1 renal, 1 oat-cell, 1 thyroid). The presence of tumour cells in the fluid was checked by an independent pathologist (Dr J. Davis). None of the patients had been treated during the 4 weeks before the assay.

*Cells.*—Fluid was centrifuged at 600 *g* for 10 min and the pellet resuspended in McCoy's 5A medium containing 10% foetal calf serum. Cells were washed twice in this medium and counted in a haemacytometer. Viable nucleated counts (trypan blue) were routinely more than 90% (except in Patient 5, in whom viability was less than 30%).

*Adherence/phagocytic depletion.*—Cells were incubated overnight at 37°C in a humidified atmosphere of 7·5% $CO_2$ in air in 150mm plastic dishes at a cell concentration of $10^6$ cells/ml in McCoy's 5A containing 10% FCS (10 ml/dish). Non-adherent cells were removed and the adherent layer washed twice with 5ml volumes of McCoy's 5A plus 10% FCS. The washings were pooled with the non-adherent fraction. The washed adherent layer was then removed by mechanical means with a rubber policeman. In cases where the adherent cells were to be used as a feeder monolayer, the adherence procedure was performed in 35mm plastic dishes which were subsequently used for cell culture without removal of the adherent layer. In such experiments, adherent cell layers were prepared with serial increases in the number of cells permitted to adhere ($10^4$–$10^7$). As determined directly by microscopy, the number of cells adhering in any experiment was found to be a constant percentage of the number of cells plated from any one patient for the adherence procedure (Fig. 1). In view of this linearity, experiments were described by the number of cells plated, not the number of adherent cells. This was essential because of the time required for staining and counting the adherent cells.

To remove phagocytic cells from the non-adherent tumour-cell suspension, the non-adherent cells were then incubated at a concentration of $10^6$ cells/ml in McCoy's 5A+10% FCS containing 40 mg dry-heat-sterilized carbonyl iron/$10^7$ cells in 250ml Falcon flasks. After an incubation at 37°C for 45 min in a shaking water bath, the flasks were placed flat on a magnet and the supernatant cells carefully decanted. These cells were subjected to this magnetic selection procedure until all iron and iron-laden phagocytic cells had been removed (usually 3 times) as checked by microscopy. The supernatant cells were then washed once in McCoy's 5A+10% FCS.

*Cell morphology and functional assessment.*—Differential counts of cell fractions were made on slides prepared with a Shandon cytocentrifuge and stained by the Papanicolaou (1954), Wright–Giemsa and nonspecific esterase (Yam *et al.*, 1971) methods. In 6 cases (Pts 4, 10d, 28, 29, 30 and 31) the macrophage component of differentials was confirmed by assessment of latex-particle ingestion. Cells ($5 \times 10^6$/ml) were incubated at 37°C for 30 min with $1{\cdot}5 \times 10^7$ polystyrene latex beads (1·101 diameter, Sigma) spread on slides, air-dried and stained with Wright's stain, and latex incorporation assessed microscopically. Positive cells were those containing 4 or more particles. In 3 cases (Pts 9, 13 and 20), cellular capacity for prostaglandin synthesis was tested: $10^6$ cells/ml in McCoy's 5A+10% FCS were incubated overnight at 37°C in a humidified atmosphere of 7·5% $CO_2$ and air, the supernatants collected by centrifugation, and prostaglandin $E_2$ and $F_{2\alpha}$ assessed by radioimmunoassay (Sigma) (Jaffe & Behrman, 1974).

*Cell culture.*—A standard two-layer agar technique was used with the enrichments described by Hamburger & Salmon (1977). No conditioned medium was present. Briefly, an underlayer of 0·5% agar in enriched McCoy's 5A medium containing 10% heat-inactivated foetal calf serum (FCS) was prepared (1 ml in a 35mm plastic Petri dish). These underlayers were in some instances poured over an adherent monolayer of cells on the surface of the dish. Cells to be tested for colony formation were suspended in a plating layer of 0·3% agar in enriched CMRL 1066 medium with 15% heat-inactivated horse serum and routinely plated at a concentration of $5 \times 10^5$ cells/ml in a 1ml plating layer above the feeder layer. For the studies of indomethacin, the appropriate concentration of the agent was incorporated into the feeder layer at the time the cultures were initiated. Cultures were incubated at 37°C in a 7·5% $CO_2$ humidified atmosphere and scored with an inverted microscope at 100× and 200×, 10–14 days after plating. Aggregates of 30 or more tumour cells were considered colonies. The neoplastic origin of the colonies was established by cytogenetics (Hamburger *et al.*, 1978) immunofluorescence (Buick *et al.*, 1979*b*) and by morphological criteria using a dried-agar-layer technique (Salmon & Buick, 1979) with specialized stains.

### RESULTS

### *Clonogenicity and morphological assessment of unfractionated cells*

The results of morphological assessment and determination of clonogenic plating efficiency in 38 unfractionated malignant effusions from 31 patients are shown in Table 1a. All patient cell populations except that from Pt 5 had viability (as assessed by trypan blue exclusion) of >90%. The values shown for macrophage percentage are those provided by analysis of Wright–Geimsa- and Papanicolaou-stained slides. Latex-particle ingestion in 6 patients provided consistently lower values of macrophage percentage (range: 0·5%, Pt 31 to 15·0%, Pt 29). On the other hand the percentage of nonspecific esterase-positive cells consistently overestimated (by 5–25%) the number of macrophages assessed by morphological analysis. Colony formation and cloning efficiency varied considerably, from 0 to 11,386 colonies/$5 \times 10^5$ cells and from $< 2 \times 10^{-6}$, $3{\cdot}2 \times 10^{-2}$/tumour cell respectively. Separate analysis of ovarian (n = 20) or breast (n = 11) carcinoma samples showed that the two groups were not significantly different with respect to cellular differential, or to clonogenic efficiencies (Table Ib). The higher mean clonogenic efficiency of ovarian effusions is due to the high clonogenicity of one sample (Pt 21b). The results of Spearman rank-correlation analysis of colony formation and cloning efficiency (CE) against each of the cell types of the effusion are shown in Table II. For all 37 effusions, only tumour cells showed a significant correlation with clonogeneity. Granulocytes or mesothelial cells were not analysed statistically because of the large number of samples not filtrated by these cells. When the 20 ovarian carcinoma effusions were analysed separately, a correlation was noted between plating efficiency and tumour-cell percentage (significant at the 1% level) and a negative correlation between clonogenicity and lymphoid percentage (significant at the 5% level).

TABLE 1a.—*Malignant effusion characteristics: cloning efficiency and morphological differentials*

| | | | | | Differential (%) | | | | |
|---|---|---|---|---|---|---|---|---|---|
| Pt | Tumour* | Fluid (ascites or pleural) | Unfractionated colonies/ $5 \times 10^5$ cells | CE (colonies/ $10^6$ tumour cell) | Macrophage/ monocyte | Tumour | Lymphocyte/ Lymphoblast | Mesothelial | Granulocyte |
| 1 | BR | P | 20 | 670 | 7·5 | 6 | 85 | 0 | 1·5 |
| 2 | CO | A | 229 | 3050 | 15 | 15 | 65 | 0 | 5 |
| 3 | OV | A | 0 | <17 | 2 | 12 | 85·5 | 0 | 0·5 |
| 4 | BR | P | 60 | 203 | 12 | 59 | 28 | 1 | 0 |
| 5 | THY | P | 281 | 1120 | 4·5 | 50 | 25 | 0 | 15·5 |
| 6 | OV | A | 0 | <19 | 37 | 10·5 | 51·5 | 1 | 0 |
| 7 | OV | A | 0 | <200 | 12 | 1 | 60 | 27 | 0 |
| 8a | CO | A | 229 | 2080 | 1 | 22 | 41 | 36 | 0 |
| 8b | CO | A | 27 | 540 | 2 | 10 | 85 | 3 | 0 |
| 9 | OV | A | 0 | <2 | 2 | 92 | 6 | 0 | 0 |
| 10a | BR | A | 144 | 400 | 16 | 72 | 6 | 3 | 3 |
| 10b | BR | A | 24 | 00 | 13·5 | 80 | 5·5 | 0 | 1 |
| 10c | BR | A | 250 | 505 | 1 | 99 | 0 | 0 | 0 |
| 10d | BR | A | 0 | <2 | 1 | 99 | 0 | 0 | 0 |
| 11 | CO | A | 65 | 260 | 5 | 50 | 9 | 0 | 36 |
| 12a | OV | A | 182 | 1650 | 16 | 22 | 40 | 0 | 22 |
| 12b | OV | A | 85 | 215 | 13 | 79 | 3·5 | 4·5 | 0 |
| 12c | OV | A | 381 | 1120 | 29 | 68 | 2 | 1 | 0 |
| 13 | OV | A | 80 | 239 | 11 | 67 | 19 | 0 | 3 |
| 14 | OV | A | 285 | 864 | 0 | 66 | 32 | 1 | 1 |
| 15 | OV | A | 36 | 176 | 8 | 41 | 30 | 0 | 21 |
| 16 | RE | P | 30 | 77 | 2 | 78 | 18 | 2 | 0 |
| 17 | OV | A | 19·5 | 122 | 10 | 32 | 24 | 2 | 32 |
| 18 | OV | A | 26 | 242 | 13·5 | 21·5 | 44·5 | 0 | 24·5 |
| 19 | OV | P | 901 | 2310 | 2 | 78 | 20 | 0 | 0 |
| 20 | OV | A | 29 | 69 | 3 | 84 | 0 | 13 | 0 |
| 21a | OV | A | 7·5 | 23 | 22 | 66 | 8 | 0 | 4 |
| 21b | OV | A | 11,386 | 32000 | 4 | 71 | 22 | 3 | 0 |
| 22 | OV | A | 733 | 2870 | 17 | 51 | 27 | 0 | 5 |
| 23 | BR | P | 106 | 424 | 42 | 50 | 8 | 0 | 0 |
| 24 | BR | A | 3 | 13 | 12 | 47·5 | 20 | 14·5 | 6 |
| 25 | OV | A | 83 | 205 | 2 | 81 | 2 | 14 | 1 |
| 26 | OC | P | 0 | <100 | 8 | 2 | 84 | 2 | 4 |
| 27 | BR | P | 0 | <100 | 2 | 2 | 12 | 0 | 84 |
| 28 | OV | A | 0 | <8 | 11 | 24 | 63 | 1 | 1 |
| 29 | OV | A | 13·5 | 270 | 21 | 10 | 30 | 0 | 37 |
| 30 | BR | P | 0 | <67 | 8 | 3 | 8 | 0 | 81 |
| 31 | BR | P | 0·5 | 100 | 1 | 1 | 96 | 0 | 2 |

* BR: breast; CO: colon OV: ovary; THY: thyroid; RE: renal; OC oat cell.

TABLE 1b.—*Clonogenicity and differential analysis of malignant effusions: median and mean values*

| | CE | | Tumour-cell % | | Macrophage % | | Lymphocyte % | |
|---|---|---|---|---|---|---|---|---|
| Sample | Median | Mean | Median | Mean | Median | Mean | Median | Mean |
| Total (n = 37) | $239 \times 10^{-6}$ | $1410 \times 10^{-6}$ | 66·0 | 48·2 | 11·0 | 11·34 | 20·0 | 27·1 |
| Ovarian (n = 20) | $239 \times 10^{-6}$ | $2130 \times 10^{-6}$ | 58·5 | 48·8 | 11·5 | 11·8 | 25·5 | 28·5 |
| Breast (n = 11) | $203 \times 10^{-6}$ | $254 \times 10^{-6}$ | 50·0 | 47·1 | 8·0 | 10·5 | 8·0 | 24·4 |

*Effects of depletion of adherent and phagocytic macrophages on colony formation by clonogenic tumour cells*

Attempts to deplete macrophages by first an adherence procedure and a subsequent magnetic depletion of phagocytic cells, produces a loss of colony-forming capacity and plating efficiency by the residual tumour cells (Table III). This was observed for all 4 types of adenocarcinoma (ovarian, breast, renal, colon) in 8/9 patients. In only 1 case (Pt 21a) was the

TABLE II.—*Spearman rank correlation coefficients of colonies/5 × 10⁵ cells or CE (colonies/tumour cell) with % tumour cells, macrophages or lymphocytes*

| | | Tumour cells | Macrophages | Lymphocytes |
|---|---|---|---|---|
| All effusions (n = 37) | Colonies/ $5 \times 10^5$ | 0·6178[1] | −0·0784 | −0·3544 |
| | CE | 0·1965 | −0·0758 | −0·0090 |
| Ovarian effusions (n = 20) | Colonies/ $5 \times 10^5$ | 0·6225[1] | −0·2015 | −0·5263 |
| | CE | 0·3518 | −0·0691 | −0·2977 |

[1] Significant at the 1% level.

clonogenic potential of the tumour cells increased by the depletion procedures. The adherent fraction in all cases was markedly reduced in colony-forming potential and plating efficiency, as determined by resuspension and plating in the standard tumour colony assay. The colony-forming capacity of the non-adherent fraction was intermediate between those of the unfractionated and the non-adherent non-phagocytic fractions. The results of morphological assessment of the cell fractions are shown in Table IV. Prostaglandin synthetic capacity of fractions from 3 effusions are shown in Table V.

FIG. 2.—Effect of autologous adherent macrophages on reconstituting ovarian tumour colony formation by enriched tumour cells in a two-layer agar system which separates the host adherent cells from the tumour cells with a barrier layer of agar. Results are mean ± s.e. of triplicate plates. (Pt 12a).

### *Stimulation of tumour colony formation from non-adherent non-phagocytic cells by adherent cells*

Fig. 2 shows the effect of plating a constant number of macrophage-depleted

TABLE III.—*Tumour colony growth (colonies/5 × 10⁵ cells) after adherent and phagocytic fractionation procedures on tumour cell population*

| Pt | Unfractionated | Adherent | Non-adherent | Non-adherent Non-phagocytic |
|---|---|---|---|---|
| 4 | 60 ± 15 ($203 \times 10^{-6}$) | 0 (< $6 \times 10^{-6}$) | 11 ± 1 | 3 ± 1 ($7 \times 10^{-6}$) |
| 10a | 144 ($400 \times 10^{-6}$) | 14 ± 2 ($2{\cdot}7 \times 10^{-6}$) | — | 12 ± 1 ($33 \times 10^{-6}$) |
| 11 | 65 ± 10 ($260 \times 10^{-6}$ | 12 ± 1 ($42 \times 10^{-6}$) | — | 6 ± 0·05 ($16 \times 10^{-6}$) |
| 12a | 182 ± 12 ($1650 \times 10^{-6}$) | 9 ± 1 ($300 \times 10^{-6}$) | — | 57 ± 5 $639 \times 10^{-6}$) |
| 12c | 85 ± 10 | 0 | — | 16 ± 2 |
| 15 | 36 ± 5 ($176 \times 10^{-6}$) | 0 (< $5{\cdot}0 \times 10^{-6}$) | — | 9 ± 1 ($30 \times 10^{-6}$) |
| 17 | 19·5 ± 2 | 0 | 17 ± 1 | 12 ± 1 |
| 20 | 29 ± 2 ($69 \times 10^{-6}$) | 0 (< $3 \times 10^{-6}$) | 11 ± 1 | 0 (< $2 \times 10^{-6}$) |
| 21a | 7·5 ± 0·5 ($230 \times 10^{-6}$) | 104 ± 1 ($290 \times 10^{-6}$) | — | 579 ± 19 ($1600 \times 10^{-6}$) |

(In parentheses, CE (colonies/tumour cell).)

TABLE IV.—*Morphological assessment of fractionated malignant effusions*

| Pt | Fraction | Cell type (%) Macrophage/ monocyte | Lymphocyte/ lymphoblast | Tumour | Mesothelial | Granulocyte |
|---|---|---|---|---|---|---|
| 4 | Unf. | 12 | 28 | 59 | 1 | 0 |
| | NA/NP | 2 | 15 | 82 | 1 | 0 |
| | Adh. | 23 | 43 | 34 | 0 | 0 |
| 10a | Unf. | 16 | 6 | 72 | 3 | 3 |
| | NA/NP | 5 | 9 | 73 | 13 | 0 |
| | Adh. | 42 | 7 | 51 | 0 | 0 |
| 11 | Unf. | 5 | 9 | 50 | 0 | 36 |
| | NA* | 1 | 15 | 73 | 0 | 11 |
| | Adh. | 18 | 4 | 57 | 4 | 17 |
| 12a | Unf. | 16 | 40 | 22 | 0 | 22 |
| | NA/NP | 8 | 34 | 18 | 0 | 40 |
| | Adh. | 53 | 37 | 6 | 0 | 4 |
| 15 | Unf. | 8 | 30 | 41 | 0 | 21 |
| | NA/NP | 2 | 10 | 60 | 0 | 28 |
| | Adh. | 19 | 29 | 42 | 0 | 10 |
| 20 | Unf. | 3 | 0 | 84 | 13 | 0 |
| | NA/NP | 2 | 0 | 97 | 1 | 0 |
| | Adh. | 18 | 0 | 63 | 18 | 1 |
| 21a | Unf. | 22 | 8 | 66 | 0 | 4 |
| | NA/NP | 0 | 12 | 86 | 2 | 0 |
| | Adh. | 25 | 5 | 71 | 0 | 0 |
| 13 | Unf. | 11 | 19 | 67 | 0 | 3 |
| | NA/NP | 1 | 10 | 86 | 1 | 1 |
| | Adh. | 27 | 61 | 11 | 0 | 1 |
| 9 | Unf. | 2 | 6 | 92 | 0 | 0 |
| | NA/NP | 0 | 4 | 96 | 0 | 0 |
| | Adh. | 2 | 1 | 97 | 0 | 0 |

* Phagocytosis procedure not done.

ovarian adenocarcinoma cells (Pt 12a) over underlayers containing increasing numbers of autologous adherent cells. A dose-dependent increase in colony formation was seen, with an optimum when $10^6$ cells were used to prepare the adherent layer. Physical separation of the purified tumour cells from the feeder layer of adherent macrophages by the agar underlayer provided direct proof that modulation of tumour growth was attributable to diffusible factors. Increasing numbers of adherent cells plated in excess of this figure decreased the number of tumour colonies. The basic pattern of reconstitution of tumour colony-forming ability was similar for all the ovarian, breast, and colon adenocarcinoma patients tested. Table VI presents reconstituted data for all 9 patients. The actual number of adherent cells in each experiment varied with the differing percentage of adherent cells in each effusion fluid.

When more than optimum numbers of adherent cells were used, there was always inhibition of tumour colony growth. Stimulation of colony formation could be achieved by incorporating harvested adherent cells within the underlayer (results not shown). In one case (Pt 21a) reconstitution decreased the colony formation. Attempts to reproduce these results with

TABLE V.—*Prostaglandin production by cells fractionated from malignant effusions* ($ng/ml/10^6$ *cells in* 18 *h*)

| Fraction | $PGE_2$ Pt 13 | $PGE_2$ Pt 20 | $PGF_{2\alpha}$ Pt 9 | $PGF_{2\alpha}$ Pt 13 | $PGF_{2\alpha}$ Pt 20 |
|---|---|---|---|---|---|
| Unf. | 31·0 | 0·56 | 9·4 | 20·0 | 0·64 |
| NA | 1·3 | 0·35 | — | 3·2 | 0·52 |
| NA/NP | 0·31 | 0·15 | 2·6 | 2·1 | 0·17 |
| Adh. | 87·0 | 6·8 | 13·5 | $>$33·0 | 6·7 |

FIG. 3.—Effect of incorporation of indomethacin in (A) the feeder layer of a reconstitution experiment (Pt 20) or (B) in the feeder layer of the basic system with unfractionated clonogenic cells (Pts 21b (●—●), 8a (□), 10c (▲), 13 (■)) and effusion cells from a patient with pancreatic cancer (△). (B) Points are mean of duplicate plates and expressed as percentage of control plates. The reconstitution experiment (A) was conducted with a feeder layer prepared over $3{\cdot}6 \times 10^5$ adherent cells and the plating layer containing $5 \times 10^5$ non-adherent/non-phagocytic cells.

conditioned media derived from adherent cells have so far been unsuccessful.

*Effect of indomethacin on tumour colony growth*

Fig. 3b displays log–dose response curves for the action of indomethacin on the clonogenic potential of 6 unfractionated effusions, and Fig. 3a a similar analysis of sensitivity of a reconstitution experiment (Pt 20). Response occurs at lower concentration in the reconstitution system than in the unfractionated system.

DISCUSSION

The experiments described here analyse cell–cell interaction involved in the clonogenicity of tumour cells in malignant effusions of patients with epithelial cancer. In the total population studied, marked variation in clonogenicity and plating efficiency was seen when unfractionated cells from effusions were plated in a two-layer agar system. Correlation analysis (Table II) of this data with cellular differentials indicate that colony formation is positively correlated ($r = 0{\cdot}6179$, $P < 0{\cdot}01$) with the percentage of tumour cells. There is no statistically significant correlation between cloning efficiency (colonies/tumour cell) and the percentages of either tumour cells, macrophages or lymphoid cells in the malignant effusion. Separate analysis of the 20 ovarian effusions demonstrated a similar correlation between colony formation and tumour-cell percentage, significant at the 1% level ($r = 0{\cdot}6225$). The negative correlations between colony formation and lymphoid-cell percentage could be accounted for by the negative correlation between tumour cells and lymphoid cells ($r = -0{\cdot}8722$, $P < 0{\cdot}01$, $n = 38$).

The fractionation experiments described in Table III provide direct evidence that in the majority of cases adherent and phagocytic cells are required for optimal *in vitro* tumour-colony growth in 4 different types of carcinoma. In one case, however, the reverse situation occurred. Results in 8 patients show that the loss in tumour-colony formation was not

TABLE VI.—*Reconstitution of colony formation*

| No. of cells plated for adherent layer | Colonies/$5 \times 10^5$ NA/NP cells plated | | | | | | | |
|---|---|---|---|---|---|---|---|---|
| Pt | None | $10^4$ | $5 \times 10^4$ | $10^5$ | $5 \times 10^5$ | $10^6$ | $5 \times 10^6$ | $10^7$ |
| 4 | 3 | 23 | 15 | 17 | 11 | 2 | — | — |
| 10a | 12 | 32 | 22 | 30 | 18 | 16 | 4 | — |
| 11 | 6 | 10 | 10 | 15 | — | 12 | 88 | — |
| 12a | 57 | — | 48 | 45 | 66 | 125 | — | 32 |
| 12c | 16 | 12 | — | 64 | — | 33 | — | — |
| 15 | 9 | 19 | — | 43 | — | 99 | — | — |
| 19 | 12 | — | 15 | 39 | 33 | 10 | — | — |
| 20 | 0 | 0 | — | 6 | — | 12 | — | 4 |
| 21a | 579 | 484 | — | 256 | — | 242 | — | — |

attributable to depletion of clonogenic tumour cells during the fractionation procedures, but rather to a loss of adherent cells required for stimulation of tumour growth through cooperative interaction. The results of morphological analysis (Table IV) and measurements of prostaglandin synthetic capacity (Table V) are indicative of an enrichment of monocyte/macrophages in the adherent fractions. It is possible that other cells (polymorphonuclear leucocytes and certain tumour cells) release prostaglandins under these conditions, and that prostaglandin release may be altered by the adherence procedure. However, it is clear that morphological assessment alone does not completely account for the enrichments and depletions observed functionally in the clonogenicity and prostaglandin-synthetic data.

To rule out the possibility that cell death in the clonogenic fraction was contributing to the result of adherence-depletion studies, we designed reconstitution procedures which would define the stoichiometry of the requirement for adherent cells. When adherence/phagocytosis-depleted ovarian, breast or colon adenocarcinoma cells were plated in the upper layer of a two-layer agar system which physically separated them from the monolayer of adherent cells, colony growth could be reconstituted. Dose dependency was observed between adherent host cells and enriched tumour cells, each patient demonstrating increasing number of tumour colonies with increasing number of adherent cells until an optimum reconstitution had been achieved, above which additional adherent cells appeared inhibitory (Fig. 2; Table VI). Inasmuch as no cellular contact occurred between the adherent cells and clonogenic tumour cells, cell–cell interaction had to be mediated *via* a diffusible substance. The optimum number of adherent cells varied between patients. No immediate correlation is apparent between patient characteristics and this ratio; however, the ratio could also be highly dependent on the proportion of cells that survived the adherence procedure and remained functionally active on the plate. While this appeared to vary from patient to patient, the proportion was linear at varying cell concentrations for any one patient (*e.g.* Fig. 1). Morphological and functional assessment of the fractions involved in the reconstitution experiments supported the role of the macrophage as the operative cell in this interaction. We cannot exclude the possibility that a subpopulation of lymphoid, granulocytic or tumour cells which also adhere might play an accessory role in this growth-stimulatory process. It is possible that tumour cells produce colony-stimulating activity (CSA) (Okabe *et al.*, 1978) which has been shown to be capable of influencing prostaglandin E production (Kurland *et al.*, 1979) and, furthermore, contaminating polymorphonuclear leucocytes can affect CSA by release of lactoferrin (Broxmeyer *et al.*, 1978). However, it has previously been reported that phagocytic depletion alone (which would not remove lymphoid cells) markedly reduces tumour-colony growth (Hamburger *et al.*, 1978) reinforcing the central regulatory role of the macrophage. Studies of unfractionated and reconstituted effusion cell populations showed inhibition of tumour-colony growth by addition of indomethacin (Fig. 3). Further fractionation studies and investigations with synthetic prostaglandins will be required to determine the relationship between inhibition of prostaglandin synthesis and inhibition of tumour-colony formation. Such clonogenicity is undoubtedly based on complex growth requirements and thus we are reluctant to assign a central role of macrophage-derived prostaglandin in this process *in lieu* of more definite evidence.

The data which we report here indicate that cell cooperation between host and tumour populations has an important role in the determination of *in vitro* tumour-cell clonogenicity. Responsibility for such stimulatory and inhibitory effects cannot be assigned definitely on the basis of these experiments although the weight of evidence points to the monocyte/macrophage series as being operative. Since the dose response of the interaction shows stimulation at certain concentrations and inhibition at higher levels, it is not surprising that in one patient fractionation increased clonogenicity. It can be suggested that in this patient the status of the cell interaction in the unfractionated effusion was such that clonogenicity was inhibited. Removal of adherent and phagocytic cells released this inhibition. Since no obvious difference could be seen in the morphological differential of this effusion, it must be assumed that the operative stimulating and inhibiting cells in this system are probably a functionally active subpopulation of the morphologically recognizable cell types. Our morphological, phagocytic and prostaglandin-synthetic analyses permit us to identify macrophages clearly as a component of the adherent underlayers. We have not yet assessed the adherent lymphoid cells to determine whether they express markers of B- or T-cell origin.

A number of investigators have recently shown that macrophages can either inhibit or promote growth of tumour cells *in vitro* (*e.g.* Norbury, 1977; Mantovani *et al.*, 1979). Such analyses have been carried out primarily on cell lines or with transplantable animal tumours. Experimental studies demonstrating inhibition of tumour growth have frequently used much larger ratios of macrophages to tumour cells (*e.g.* 20:1 or 100:1) than we have found in malignant effusions (Table I). Stephens *et al.* (1978) have demonstrated a requirement for tumour cells in the development of macrophage colonies in agar from cells derived from Lewis lung carcinoma, and draw attention to the need to distinguish between CFU-C and tumour colonies in such systems. However, under the conditions of assay employed, and cell types involved in our study, colony formation was restricted to epithelial cells.

The analysis of controlling influences on the primary growth of clonogenic human tumour cells which we report suggest that interactions between host cells and tumour cells occur in human carcinomas, and provide supportive evidence implicating the macrophage as a potential source of stimulation to tumour growth.

This work was supported in part by Grants CA-21839 and CA-17094 and CA-23074 from the United States Public Health Service, Bethesda, Maryland 20014.

We thank Professor J. Davis of the Department of Pathology for his assistance with the morphological analysis and review of tumour cytology samples, and Yvette Frutiger and Barbara Soehnlen for technical assistance.

### REFERENCES

BROXMEYER, H. E., SMITHYMAN, A., EGER, R. R., MEYERS, P. A. & DESOUSA, M. (1978) Identification of lactoferrin as the granulocyte-derived inhibitor of colony-stimulating activity production. *J. Exp. Med.*, **148**, 1952.

BUICK, R. N., STANISIC, T., FRY, S. E., SALMON, S. E., TRENT, J. M. & KRASOVICH, P. (1979*a*) Development of a methylcellulose/agar clonogenic assay for cells in transitional cell carcinoma of the human bladder. *Cancer Res.*, **39**, 5051.

BUICK, R. N., FRY, S. E. & SALMON, S. E. (1979*b*) Tumor progenitor cell assays: Application of techniques to colon cancer. *Cancer*, **45**, 1238.

EVANS, R. (1976) Tumor macrophages in host immunity to malignancies. In *The Macrophage in Neoplasia*. Ed. Finck. New York: Academic Press. p. 27.

FIDLER, I. J., BRODEV, R. S. & BECK-NIELSEN, S. (1974) *In vitro* immune stimulation–inhibition to spontaneous canine tumors of various histologic types. *J. Immunol.*, **112**, 1051.

HAMBURGER, A. W., SALMON, S. E., KIM, M. B. & 4 others (1978) Direct cloning of human ovarian carcinoma cells in agar. *Cancer Res.*, **38**, 3438.

HAMBURGER, A. W. & SALMON, S. E. (1977) Primary bioassay of human tumor stem cells. *Science*, **199**, 461.

HOLDEN, H. T., HASKILL, J. S., KIRCHNER, H. & HERBERMAN, R. B. (1976) Two functionally distinct anti-tumor effector cells isolated from primary murine sarcoma-virus induced tumors. *J. Immunol.*, **117**, 440.

JAFFE, B. M. & BEHRMAN, H. R. (1974) In *Methods of Hormone Radioimmunoassay*. New York: Academic Press. p. 19.

KURLAND, J. I., PELUS, L. M., RALPH, P., BOCKMAN, R. S. & MOORE, M. A. S. (1979) Induction of prostaglandin E and synthesis in normal and neoplastic macrophages: Role for colony-stimulating factor(s) distinct from effects on myeloid progenitor cell proliferation. *Proc. Natl Acad. Sci. U.S.A.*, **76**, 2326.

LIGHT, R. W., EROZAN, Y. S. & BALL, W. C., JR (1973) Cells in pleural fluid. *Arch. Int. Med.*, **132**, 854.

MANTOVANI, A., PERI, G., POLENTARUTTI, N., BOLIS, G., MANGIONI, C. & SPREAFICO, F. (1979) Effects on *in vitro* tumor growth of macrophages isolated from human ascitic ovarian tumors. *Int. J. Cancer*, **23**, 157.

NORBURY, K. C. (1977) *In vitro* stimulation and inhibition of tumor cell growth mediated by different lymphoid cell populations. *Cancer Res.*, **37**, 1408.

OKABE, T., SATO, N., KONDO, Y. & 4 others (1978) Establishment and characterization of a human cancer cell line that produces human colony-stimulating factor. *Cancer Res.*, **38**, 3910.

PAPANICOLAOU, G. N. (1954) *Atlas of Exfoliative Cytology*. Cambridge: Harvard University Press. p. 6.

PREHN, R. T. (1977) Immunostimulation of the lymphodependent phase of neoplastic growth. *J. Natl Cancer Inst.*, **59**, 1043.

SALMON, S. E. & BUICK, R. N. (1979) Preparation of permanent slides of intact soft-agar colony cultures of hematopoietic and tumor stem cells. *Cancer Res.*, **39**, 1133.

SALMON, S. E. & HAMBURGER, A. W. (1978) Immunoproliferation and cancer: a common macrophage-derived promoter substance. *Lancet*, i, 1289.

STEEL, G. G. (1977) *Growth Kinetics of Tumors*. Oxford: Clarendon Press.

STEPHENS, T. C., CURRIE, G. A. & PEACOCK, J. H. (1978) Repopulation of α-irradiated Lewis lung carcinoma by malignant cells and host macrophage progenitors. *Br. J. Cancer*, **38**, 537.

UNDERWOOD, J. C. E. (1974) Lymphoreticular infiltration in human tumors: Prognostic and biological implications: A review. *Br. J. Cancer*, **30**, 538.

WOOD, G. W. & GILLESPIE, G. Y. (1975) Studies on the role of macrophages in regulation of growth and metastasis of murine chemically induced fibrosarcomas. *Int. J. Cancer*, **16**, 1022.

YAM, L. T., LI, E. Y. & CROSBY, W. H. (1971) Cytochemical identification of monocytes and granulocytes. *Am. J. Chem. Pathol.*, **55**, 283.


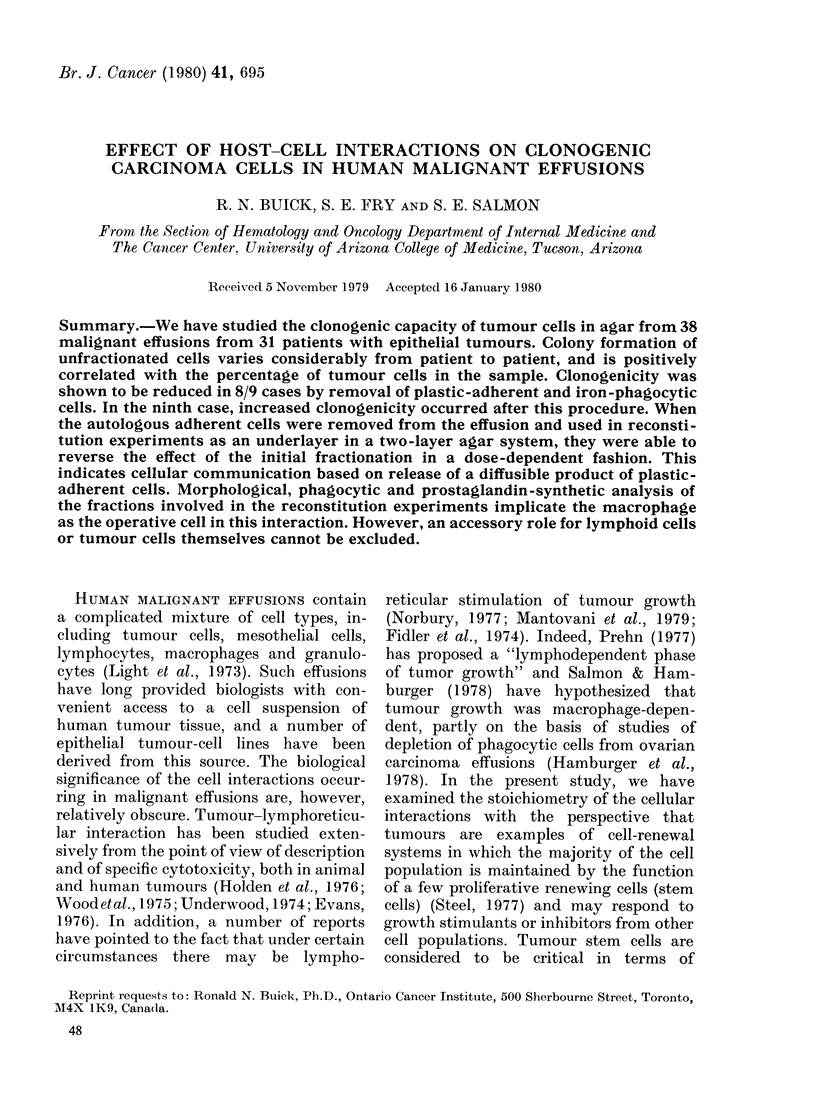

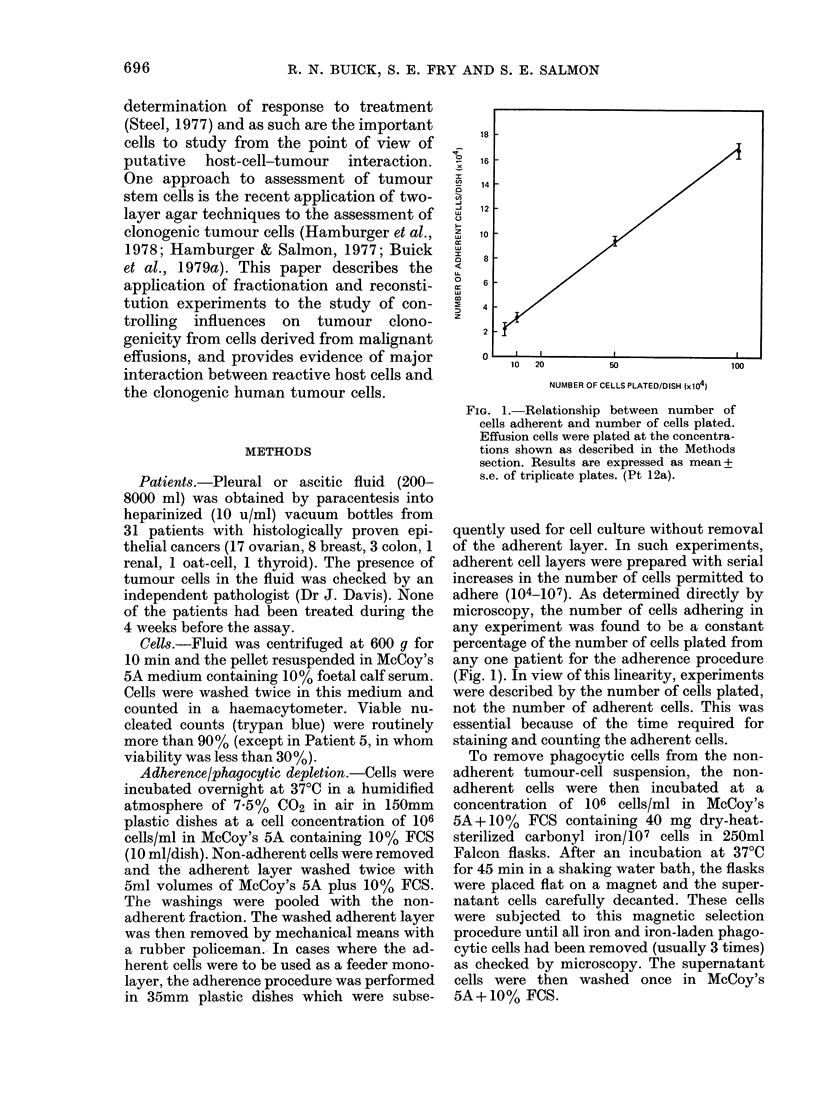

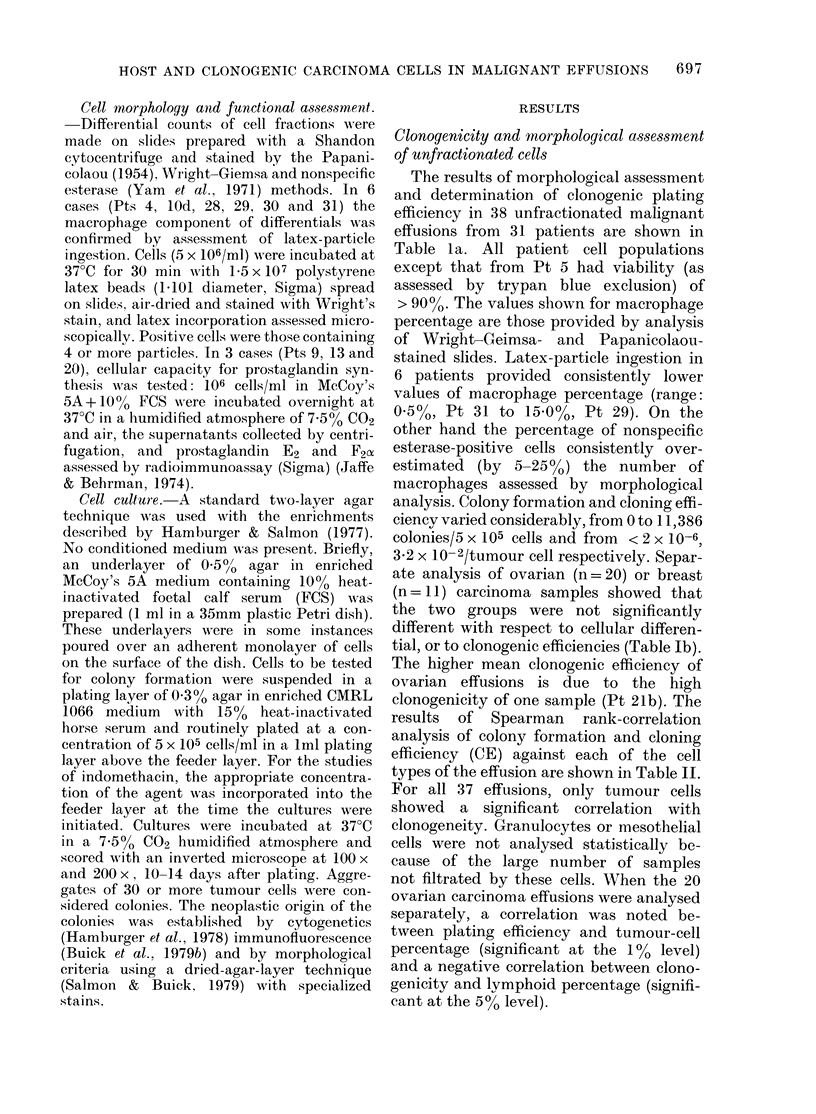

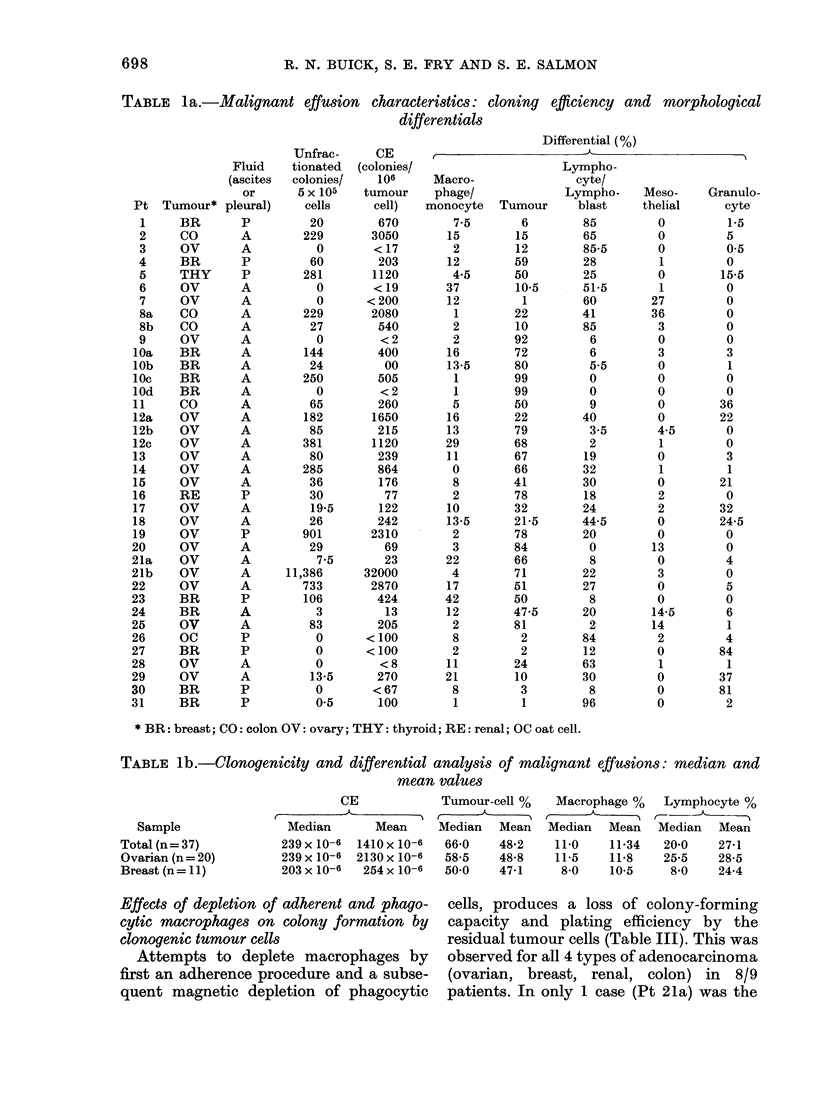

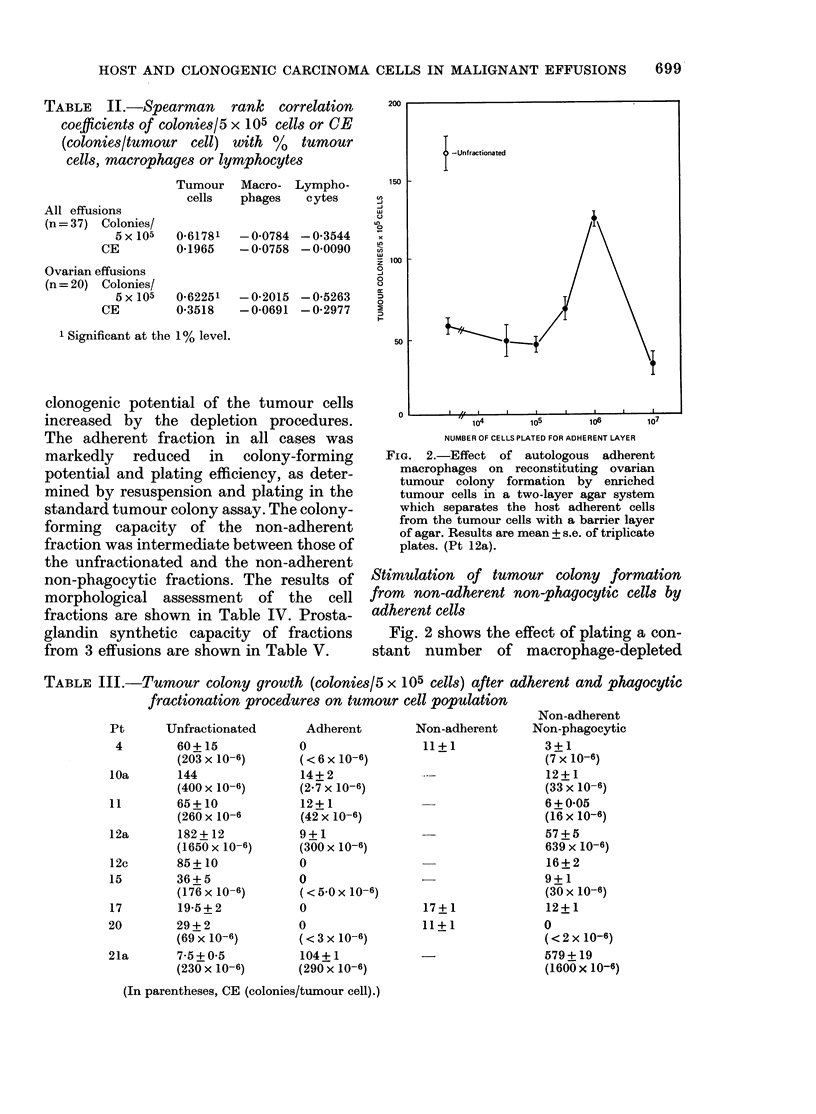

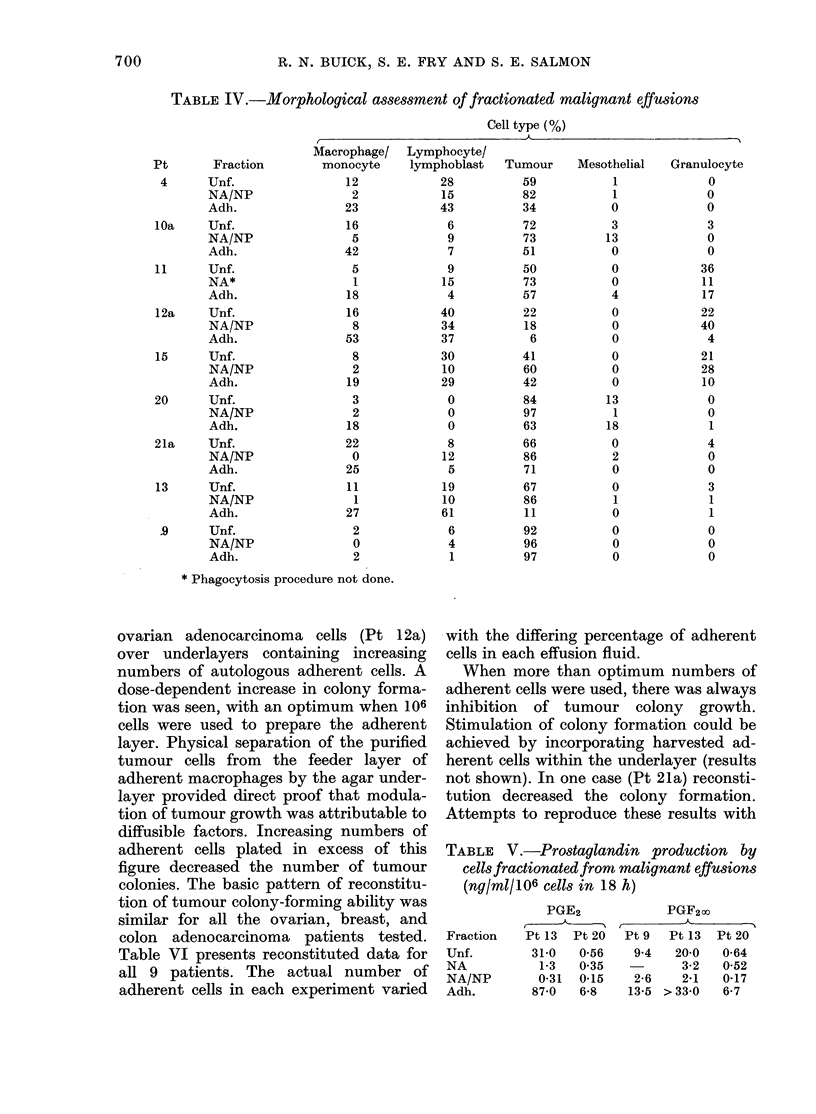

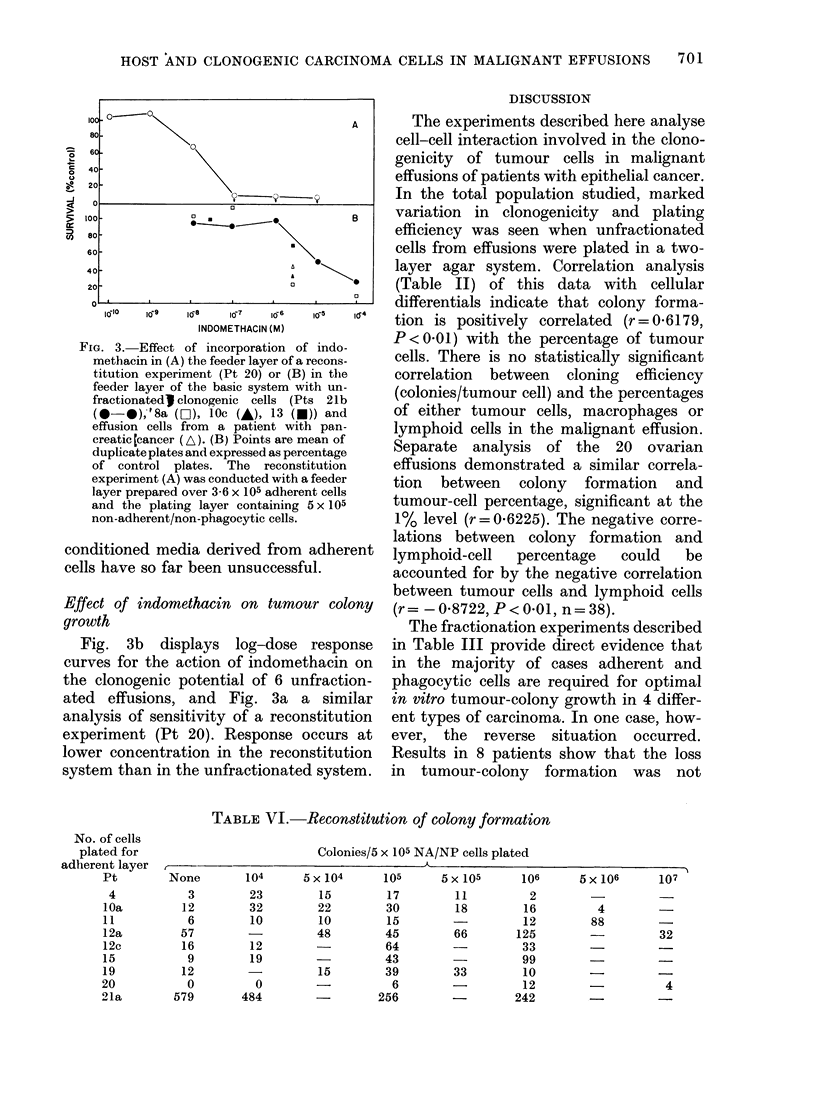

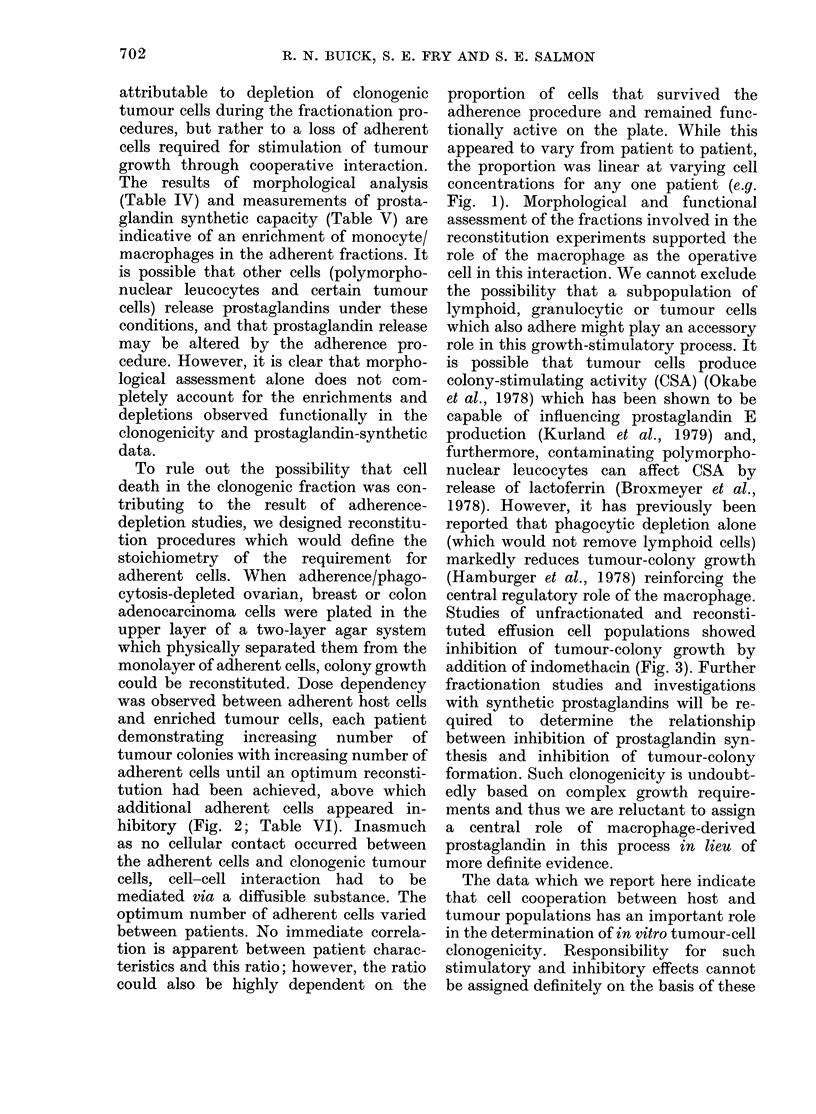

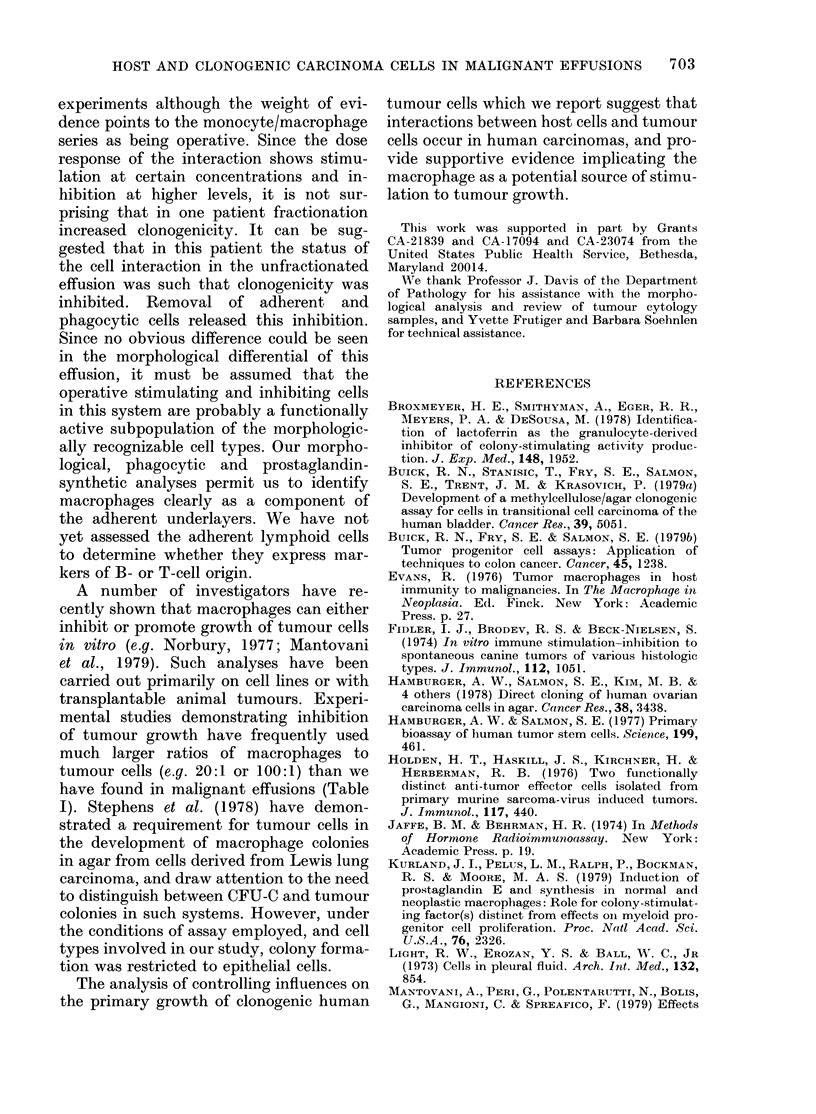

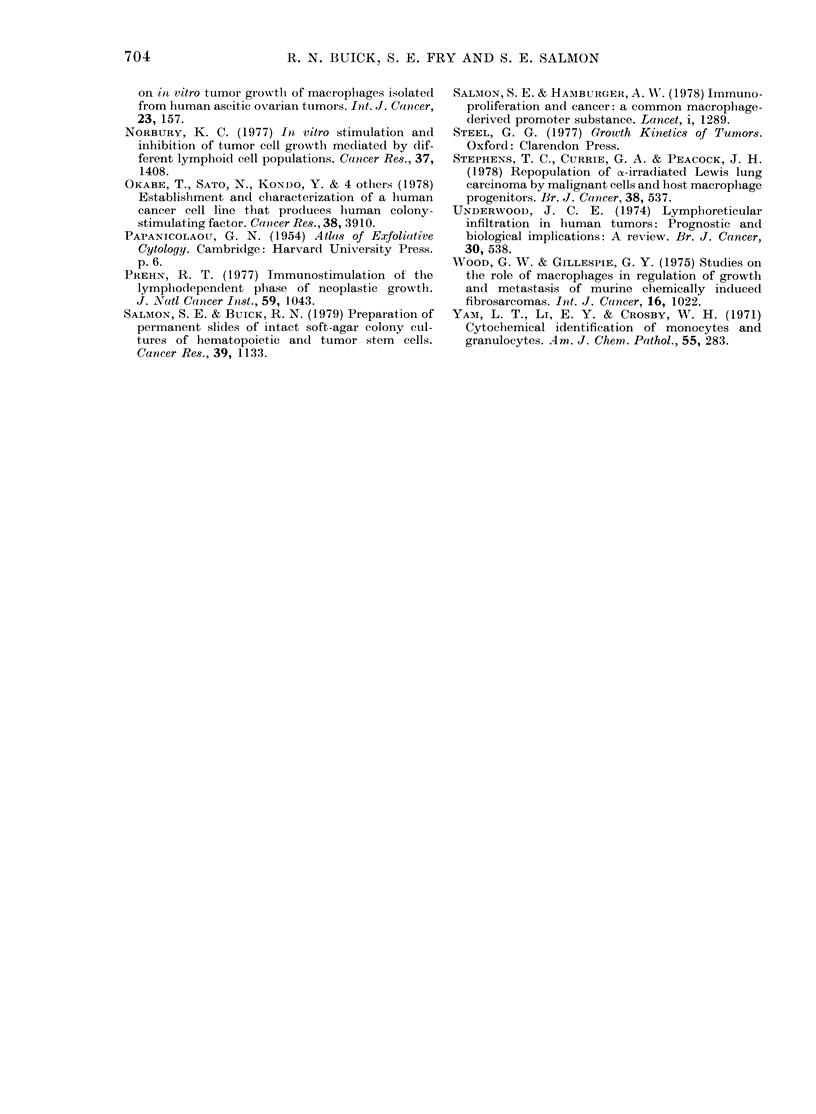


## References

[OCR_01685] Buick R. N., Fry S. E., Salmon S. E. (1980). Application of in vitro soft agar techniques for growth of tumor cells to the study of colon cancer.. Cancer.

[OCR_01678] Buick R. N., Stanisic T. H., Fry S. E., Salmon S. E., Trent J. M., Krasovich P. (1979). Development of an agar-methyl cellulose clonogenic assay for cells in transitional cell carcinoma of the human bladder.. Cancer Res.

[OCR_01696] Fidler I. J., Brodey R. S., Bech-Nielsen S. (1974). In vitro immune stimulation-inhibition to spontaneous canine tumors of various histologic types.. J Immunol.

[OCR_01702] Hamburger A. W., Salmon S. E., Kim M. B., Trent J. M., Soehnlen B. J., Alberts D. S., Schmidt H. J. (1978). Direct cloning of human ovarian carcinoma cells in agar.. Cancer Res.

[OCR_01707] Hamburger A. W., Salmon S. E. (1977). Primary bioassay of human tumor stem cells.. Science.

[OCR_01712] Holden H. T., Haskill J. S., Kirchner H., Herberman R. B. (1976). Two functionally distinct anti-tumor effector cells isolated from primary murine sarcoma virus-induced tumors.. J Immunol.

[OCR_01724] Kurland J. I., Pelus L. M., Ralph P., Bockman R. S., Moore M. A. (1979). Induction of prostaglandin E synthesis in normal and neoplastic macrophages: role for colony-stimulating factor(s) distinct from effects on myeloid progenitor cell proliferation.. Proc Natl Acad Sci U S A.

[OCR_01733] Light R. W., Erozan Y. S., Ball W. C. (1973). Cells in pleural fluid. Their value in differential diagnosis.. Arch Intern Med.

[OCR_01738] Mantovani A., Peri G., Polentarutti N., Bolis G., Mangioni C., Spreafico F. (1979). Effects on in vitro tumor growth of macrophages isolated from human ascitic ovarian tumors.. Int J Cancer.

[OCR_01748] Norbury K. C. (1977). In vitro stimulation and inhibition of tumor cell growth mediated by different lymphoid cell populations.. Cancer Res.

[OCR_01754] Okabe T., Sato N., Kondo Y., Asano S., Ohsawa N., Kosaka K., Ueyama Y. (1978). Establishment and characterization of a human cancer cell line that produces human colony-stimulating factor.. Cancer Res.

[OCR_01765] Prehn R. T. (1977). Immunostimulation of the lymphodependent phase of neoplastic growth.. J Natl Cancer Inst.

[OCR_01785] Reddy J. K., Rao M. S. (1978). Enhancement by Wy-14,643, a hepatic peroxisome proliferator, of diethylnitrosamine-initiated hepatic tumorigenesis in the rat.. Br J Cancer.

[OCR_01770] Salmon S. E., Buick R. N. (1979). Preparation of permanent slides of intact soft-agar colony cultures of hematopoietic and tumor stem cells.. Cancer Res.

[OCR_01776] Salmon S. E., Hamburger A. W. (1978). Immunoproliferation and cancer: a common macrophage-derived promoter substance.. Lancet.

[OCR_01791] Underwood J. C. (1974). Lymphoreticular infiltration in human tumours: prognostic and biological implications: a review.. Br J Cancer.

[OCR_01797] Wood G. W., Gillespie G. Y. (1975). Studies on the role of macrophages in regulation of growth and metastasis of murine chemically induced fibrosarcomas.. Int J Cancer.

[OCR_01803] Yam L. T., Li C. Y., Crosby W. H. (1971). Cytochemical identification of monocytes and granulocytes.. Am J Clin Pathol.

